# Lipoprotein Lipase Expression in Hypothalamus Is Involved in the Central Regulation of Thermogenesis and the Response to Cold Exposure

**DOI:** 10.3389/fendo.2018.00103

**Published:** 2018-03-14

**Authors:** Elise Laperrousaz, Raphaël G. Denis, Nadim Kassis, Cristina Contreras, Miguel López, Serge Luquet, Céline Cruciani-Guglielmacci, Christophe Magnan

**Affiliations:** ^1^Unité de Biologie Fonctionnelle et Adaptative, CNRS UMR 8251, Sorbonne Paris Cité, Université Denis Diderot, Paris, France; ^2^NeurObesity Group, Department of Physiology, Centro de Investigación en Medicina Molecular y Enfermedades Crónicas (CiMUS), University of Santiago de Compostela-Instituto de Investigación Sanitaria, Santiago de Compostela, Spain; ^3^CIBER Fisiopatología de la Obesidad y Nutrición (CIBERobn), Santiago de Compostela, Spain

**Keywords:** lipoprotein lipase, cold exposure, hypothalamus, brown adipose tissue, browning of white adipose tissue

## Abstract

Lipoprotein lipase (LPL) is expressed in different areas of the brain, including the hypothalamus and plays an important role in neural control of the energy balance, including feeding behavior and metabolic fluxes. This study tested the hypothesis that hypothalamic LPL participates in the control of body temperature. We first showed that cold exposure induces decreased activity and expression of LPL in the mouse hypothalamus. We then selectively deleted LPL in the mediobasal hypothalamus (MBH) through an adeno-associated virus approach in LPL-floxed mice and generated MBHΔ*^Lpl^* mice with 30–35% decrease in hypothalamic LPL activity. Results showed a decrease in body temperature in MBHΔ*^Lpl^* mice when compared with controls at 22°C. Exposure to cold (4°C for 4 h) decreased the body temperature of the control mice while that of the MBHΔ*^Lpl^* mice remained similar to that observed at 22°C. MBHΔ*^Lpl^* mice also showed increased energy expenditure during cold exposure, when compared to controls. Finally, the selective MBH deletion of LPL also increased the expression of the thermogenic PRMD16 and Dio2 in subcutaneous and perigonadal adipose tissues. Thus, the MBH LPL deletion seems to favor thermogenesis. These data demonstrate that for the first time hypothalamic LPL appears to function as a regulator of body temperature and cold-induced thermogenesis.

## Introduction

Adaptation to change in ambient temperature, especially cold exposure, is one of the mechanisms essential to the survival of mammals. This adaptation involves numerous signals of neural or hormonal origin and acute responses or acclimatization to prolonged cold exposures (1). Acute responses include vasoconstriction or shivering. The hypothalamus is a key area of central nervous system (CNS) involved in the adaptation to cold exposure. Indeed, afferent signals from the skin are sensed by the preoptic area of the anterior hypothalamus. The measurement of c-fos expression also suggests a role for the dorsomedial, medial, and the ventromedial hypothalamus after cold exposure in rats (2). In addition, several studies have also highlighted a role for arcuate (ARC) or ventromedial (VMH) nuclei in this adaptation (3, 4). Peripheral adaptation to cold exposure involves the activation of the sympathetic nervous system (SNS) and hypothalamic–pituitary–thyroid axis (5, 6), including an increase in type 2 iodothyronine deiodinase (Dio2) expression that activates thyroxine (T4) to 3,3′,5-triiodothyronine (T3) (7). These signals combine to activate catabolic pathways with release of energy substrates such as free fatty acids (FFA) into the bloodstream from white adipose tissue (WAT). FFA can then serve to fuel shivering thermogenesis in muscle (8). In contrast, triglycerides (TG) stored in the brown adipose tissue (BAT) are also mobilized as FFA *via* activation of the SNS, however, these FFA are used primarily within the BAT itself as a fuel to generate heat through uncoupling of oxidative phosphorylation (8). Furthermore, browning of WAT during cold exposure can also be an adaptive mechanism to produce thermogenesis accompanied by increased expression of beige adipocyte markers such as Dio2, PR domain containing 16 (PRDM16), cell death-inducing DNA fragmentation factor α-subunit effector 1 (Cidea), and carnitine palmitoyl transferase-1b (Cpt1b) (9–11). In Humans, short-term cooling increases serum FFA, TG, and total cholesterol concentration (12). Short-term cooling also enhances large very low-density lipoprotein, small low-density lipoprotein, and small high-density lipoprotein particle number (12). Hydrolysis of TG during cold exposure is mainly due to lipoprotein lipase (LPL) that supplies FFA for nonshivering thermogenesis. Short-term cold exposure (i.e., 4–8 h) increased LPL activity in BAT in rats (13), Djungarian hamsters (14), and mice (15). Of note, other lipases such as patatin like phospholipase domain containing 2 [Pnpla2, also known as adipose triglyceride lipase (ATGL)] and hormone-sensitive lipase (HSL) are also affected by cold exposure (16). Interestingly, this cold-induced rise of LPL activity is specific to BAT, while cold exposure decreases epididymal WAT LPL activity in rats (17). Finally, LPL is strongly expressed in the CNS especially in regions involved in the control of the energy balance (18). We have previously shown that a decrease in the expression of LPL in hippocampus (19) or hypothalamus (20) led to the development of obesity and dysregulation of energy homeostasis. Thus, it was tempting to investigate whether hypothalamic LPL was involved in regulation of body temperature depending on different ambient conditions, such as moderate hypothermia relative to thermoneutrality (22°C) or cold (4°C). The passage from 22 to 4°C represents an adaptive challenge requiring increased thermogenic needs (21). To that end, mice deficient for LPL in mediobasal (ARC + VMH) hypothalamus (MBHΔ*^Lpl^*) were generated by bilateral injections of adeno-associated virus (AAV)-cre in the mediobasal hypothalamus (MBH) of Lpl^lox/lox^ male mice, leading to a 30–35% decrease in hypothalamic LPL activity as previously published (20). The results obtained from these mice suggest that MBH LPL dampens cold-induced thermogenesis and its deletion promotes cold adaptation.

## Materials and Methods

The experimental protocol was approved by the institutional animal care and use committee of the Paris Diderot University (CEEA40) and the agreement # 03752.02 was given to the project.

### Animal Models

Ten-week-old Lpl^lox/lox^ male mice (Jackson laboratory, strain B6.129S4-Lpltm1Ijg/J, no. 006503) and wild-type littermates were used as controls. They were housed individually in stainless steel cages in a room maintained at 22 ± 1°C with lights on from 07:00 a.m. to 07:00 p.m. They were given a standard laboratory diet (proteins 19.4%, carbohydrates 59.5%, lipids 4.6%, vitamins, and minerals 16.5%) and water *ad libitum*.

### Viral Production

An AAV Cre-GFP was used in order to induce genetic recombination within the hypothalamus in Lpl^lox/lox^ mice. The plasmid CBA.nls myc Cre.eGFP expressing the myc-nls-Cre-GFP fusion protein was kindly provided by Richard Palmiter (University of Washington, Seattle, WA, USA). Adeno-associated viruses of the serotype 2/9 (AAV2/9) (6 × 10^11^ vg/ml and 1.7 × 10^8^ pi/μl) known to have a neuronal tropism (22) were produced by the viral production facility of the UMR INSERM 1089 (Nantes, France).

### Surgical and Stereotactic Procedures

At 10 weeks of age, mice were anesthetized with isoflurane and received an i.p. injection of 180 µg/kg buprenorphine hydrochloride (Axiance, Pantin, France) analgesic before being placed on a stereotaxic frame. AAVs were injected bilaterally into the MBH (stereotactic coordinates are relative to bregma: *x* ± 0.5 mm; *y* −1.64 mm; *z* −5.7 mm) at a rate of 0.20 µl/min for a total of 0.5 µl per side, in Lpl^lox/lox^ mice (MBHΔ*^Lpl^* mice) and in wild-type C57Bl6/J used as controls (MBH*^Lpl^* mice). At the end of surgical procedures, mice received an i.p. injection of 50 µg/kg ketoprofen (Mérial, Lyon, France). Intraperitoneal probes (Anipill, Caen, France) were placed in animals during a laparotomy, to measure their body temperature. Animals had 7 days postsurgery to recover.

### Indirect Calorimetry

Animals were individually housed in a cage with lights on from 7 a.m. to 7 p.m. and an ambient temperature of 22 ± 0.5 or 7°C (see Cold Exposure). All animals were acclimated to their cages for 48 h before experimental measurements. Data were collected every 15 min. Animals were analyzed for total energy expenditure (kcal/h), oxygen consumption and carbon dioxide production (VO_2_ and VCO_2_, where V is the volume), respiratory exchange rate (RER = VCO_2_/VO_2_), food intake (g), and locomotor activity (beam breaks/h) using calorimetric cages with bedding, food and water *ad libitum* (Labmaster, TSE Systems GmbH, Bad Homburg, Germany). The instrument combined a set of highly sensitive feeding and drinking sensors for automated online measurement. To allow measurement of ambulatory movements, each cage was embedded in a frame with an infrared light beam-based activity monitoring system with online measurement at 100 Hz; the detection of movement operated efficiently in both light and dark phases, allowing continuous recording. Gas ratio was determined using an indirect open-circuit calorimeter (23), which monitored O_2_ and CO_2_ concentrations by volume at the inlet ports of a tide cage with an airflow of 0.4 l/min, with regular comparisons to an empty reference cage. Total energy expenditure was calculated according to the Weir equation, using respiratory gas exchange measurements (24). The flow was previously calibrated with O_2_ and CO_2_ mixture of known concentrations (Air Liquide, S.A. France). Fatty acid oxidation was calculated from the following equation: fat ox (kcal/h) = energy expenditure × (1 − RER/0.3) according to Bruss et al. (25). Animals were monitored for body weight and composition at the beginning and end of the experiment. Data analysis was carried out with Excel XP using extracted raw values of VO_2_ consumption, VCO_2_ production (ml/h), and energy expenditure (kcal/h). Subsequently, each value was expressed either as a function of total body weight or total lean tissue mass extracted from the EchoMRI analysis.

### Cold Exposure

Mice were exposed to cold at 10 days postsurgery. For the acute cold exposure, animals were exposed to 4°C for 4 h in a refrigerated bench, closed by a Plexiglas plate. Additional mice were exposed to 7°C for 24 h in the calorimetric cages described above.

### Body Temperature Measurement

Body temperature was measured every 15 min by telemetry with intraperitoneal probes (Anipill, Caen, France). Data were collected by an electronic monitor and transferred to a computer with Anipill Software (Anipill, Caen, France) and were then extracted to an Excel XP file (Microsoft France, Issy-les-Moulineaux, France) before analysis.

### BAT Temperature Measurement

Skin temperature surrounding BAT was recorded with an infrared camera (B335: Compact-Infrared-Thermal-Imaging-Camera; FLIR; West Malling, Kent, UK) and analyzed with a FLIR-Tools-Software (FLIR; West Malling, Kent, UK) as previously described (26, 27). For each image, the area surrounding BAT was delimited and the average temperature of the skin area was calculated as the average of two pictures/animal.

### Tissue Collection

Brain tissues were dissected following the Glowinski and Iversen technique (28); hypothalamus, hippocampus, striatum, and cerebral cortex were immediately frozen. In addition, subcutaneous (WATsc) and gonadal white adipose tissue (WATgon) and intrascapular BAT were also collected and immediately frozen for further measurement of mRNA of gene of interest.

### LPL Activity Assay

Heparin-releasable LPL activity was assayed in brain regions using a Roar LPL activity assay kit (RB-LPL, Roar Biomedical, Inc.). Briefly, tissues were lysed in 500 µl of assay buffer (150 mM NaCl, 10 mM Tris, 2 mM EDTA, pH 7.4) and incubated for 45 min at 37°C with an equivalent volume of heparin (100 U/ml). After incubation, samples were centrifuged for 10 min at 3,000 *g*, and aliquots (10 µl for mice) of the aqueous phase deposited on LPL substrate emulsion in 96-well black microplates (VWR International, # 25227-304) and incubated for 1 h at 37°C. Finally, fluorescence was read using a fluorimeter (370 nm excitation/450 nm emission), and compared to a standard curve made using known concentrations of prehydrolyzed LPL substrate. LPL activity was expressed as μmoles of FFA produced per minute per gram of tissue.

### Real-Time Quantitative PCR

Total RNA was isolated from the hippocampus dissected at day 28 at the end of the experimentation using RNeasy Lipid Tissue mini kit (Qiagen, Courtaboeuf, France). Real-time quantitative PCR was carried out in a LightCycler 480 detection system (Roche, Meylan, France) using the Light-Cycler FastStart DNA Master plus SYBR Green I kit (Roche). The mRNA transcript level for each gene was normalized against the mean of 2 HKG: rpL19 and TBP, which we have previously shown to be unaffected by LPL inhibition (Table 1: primers sequences).

**Table 1:** Primers used for RT-qPCR.

Gene	Forward primers	Reverse primers
*Rpl19*	gggcaggcatatgggcata	ggcggtcaatcttcttggatt
*Tbp*	ggggagctgtgatgtgaagt	ccaggaaataattctggctca
*Abhd5*	atctttggagcccgatcct	cttctggctgatctgcatacac
*Pnpla2*	tgaccatctgccttccaga	tgtaggtggcgcaagaca
*Plin5*	Agctggagcccgtgtcta	gggcatccactgcattct
*Lipe HSL*	gcctggcaaaatctgagg	gcctccgtggatgtgaac
*Lpl*	tttgtgaaatgccatgacaag	cagatgctttcttctcttgtttgt
*CerS1*	ggagctactgcgcttacctg	ccatgcctgacctccagt
*Clock*	ccagtcagttggtccatcatt	tggctcctaactgagctgaaa
*Bmal1*	cgactcattgatgccaagac	tgcacttcattctacagaacagaga
*Cidea*	ttcaaggccgtgttaagga	cctttggtgctaggcttgg
*Cpt1b*	Ctcctttcctggctgaggt	gatctggaactgggggatct
*Prdm16*	Cctaaggtgtgcccagca	caccttccgcttttctaccc
*Dio2*	tggcatgccctgtaggtt	tgagaagtctggtacatcagcaa
*Ucp1*	ggctctgcaggagtccgaagt	ggcgtgagtgcaagaacaaaa
*Ucp2*	tttgggtttggttgtttttga	ggaggcagatgcaggtagat
*Ucp3*	ctgcctgtccatcagttcaa	ccagtcctaacaattccctaaaga
*AdR*β*3*	cagccagccctgttgaag	ccttcatagccatcaaacctg

*Rpl19, ribosomal protein L19; Tbp, TATA-box binding protein; Abhd5, α/β hydrolase domain containing 5; Pnpla2, patatin-like phospholipase domain containing 2 (ATGL); Plin5, perilipin5; Lipe HSL, lipase hormone sensitive; Lpl, lipoprotein lipase; CerS1, ceramide synthase1; Clock, clock circadian regulator; Bmal1, brain and muscle Arnt-like protein-1; CIDEA, cell death-inducing DNA fragmentation factor alpha subunit-like effector A; Cpt1b, carnitine palmitoyltransferase 1B; PRDM16, PR/SET domain 16; Dio2, type 2 iodothyronine deiodinase; UCP, uncoupling protein; AdRβ3, β3-adrenergic receptor*.

### Statistical Analysis

Data are expressed as mean ± SEM. Statistical analysis were performed using Student’s test or ANOVA followed by two-by-two Bonferroni *post hoc* test (GraphPad software). *p* < 0.05 was considered significant. Representative bar graph is expressed as mean ± SEM.

## Results

### Cold Exposure Decreases Hypothalamic LPL Activity in Control Mice

**Figure 1:**
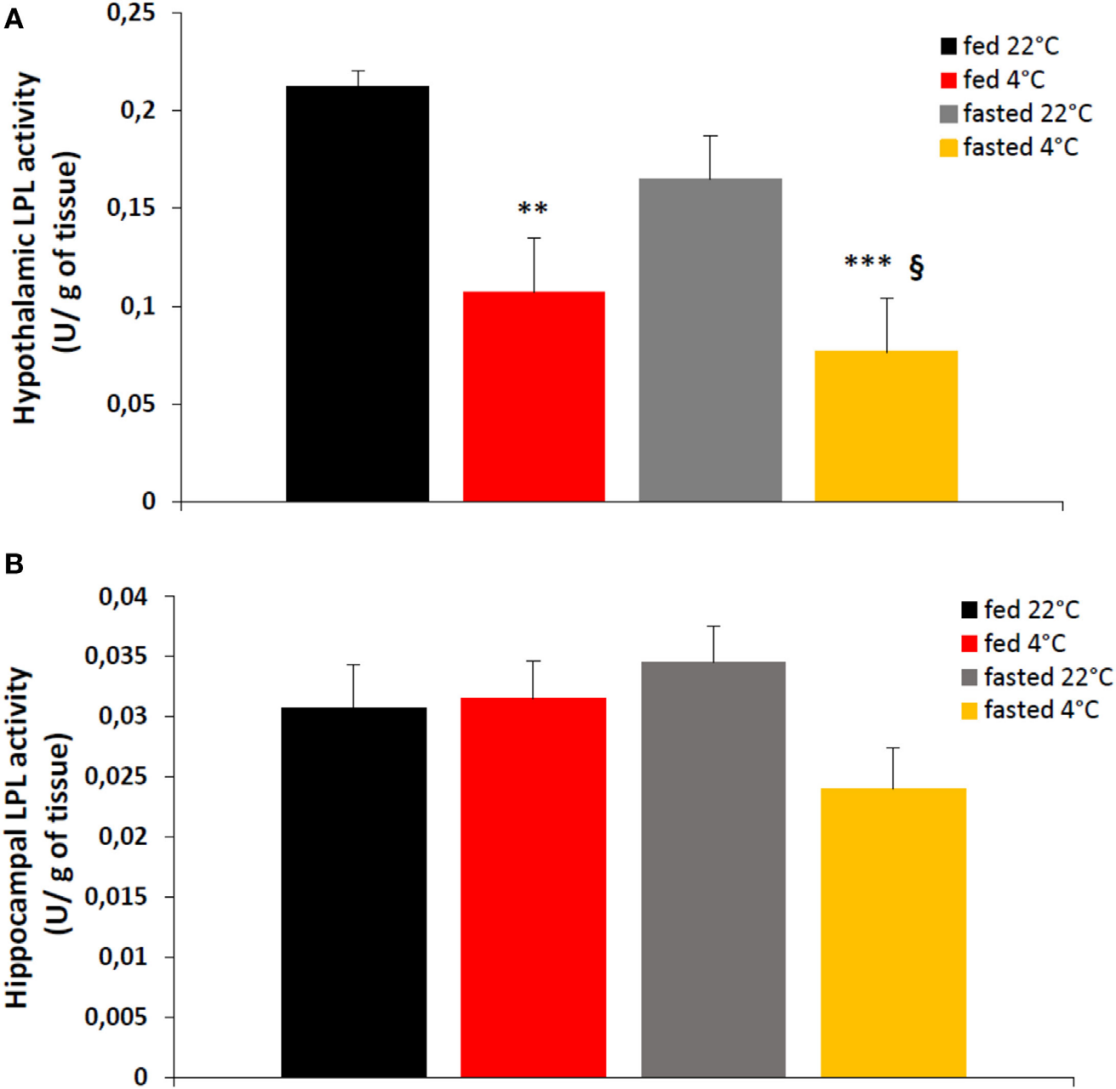
An acute cold exposure (4 h at 4°C) decreases significantly enzymatic activity of lipoprotein lipase (LPL) in hypothalamus **(A)** but not in hippocampus **(B)**. LPL activity is expressed in enzymatic units (U, μmol/min) per g of tissue. Data are mean ± SEM, *n* = 6–8 for each group. *p < 0.05 and ***p < 0.001 vs. mice “fed 22°C”, ^§^*p* < 0.05 vs. “fasted 22°C” mice.

There was no effect of nutritional status (i.e., fed or fasted) on hypothalamic LPL activity at either ambient temperatures of 22 or 4°C (Figure 1A). In contrast, in fed and fasted mice, exposure after 4 h at 4°C, there was a significant decrease of ~50% in hypothalamic LPL activity when compared with LPL activity at 22°C (Figure 1A). Of note, neither nutritional status nor ambient temperature exposure affected hippocampal LPL activity (Figure 1B).

### Cold Exposure-Induced Changes in Brain Gene Expression in Control Mice

**Figure 2:**
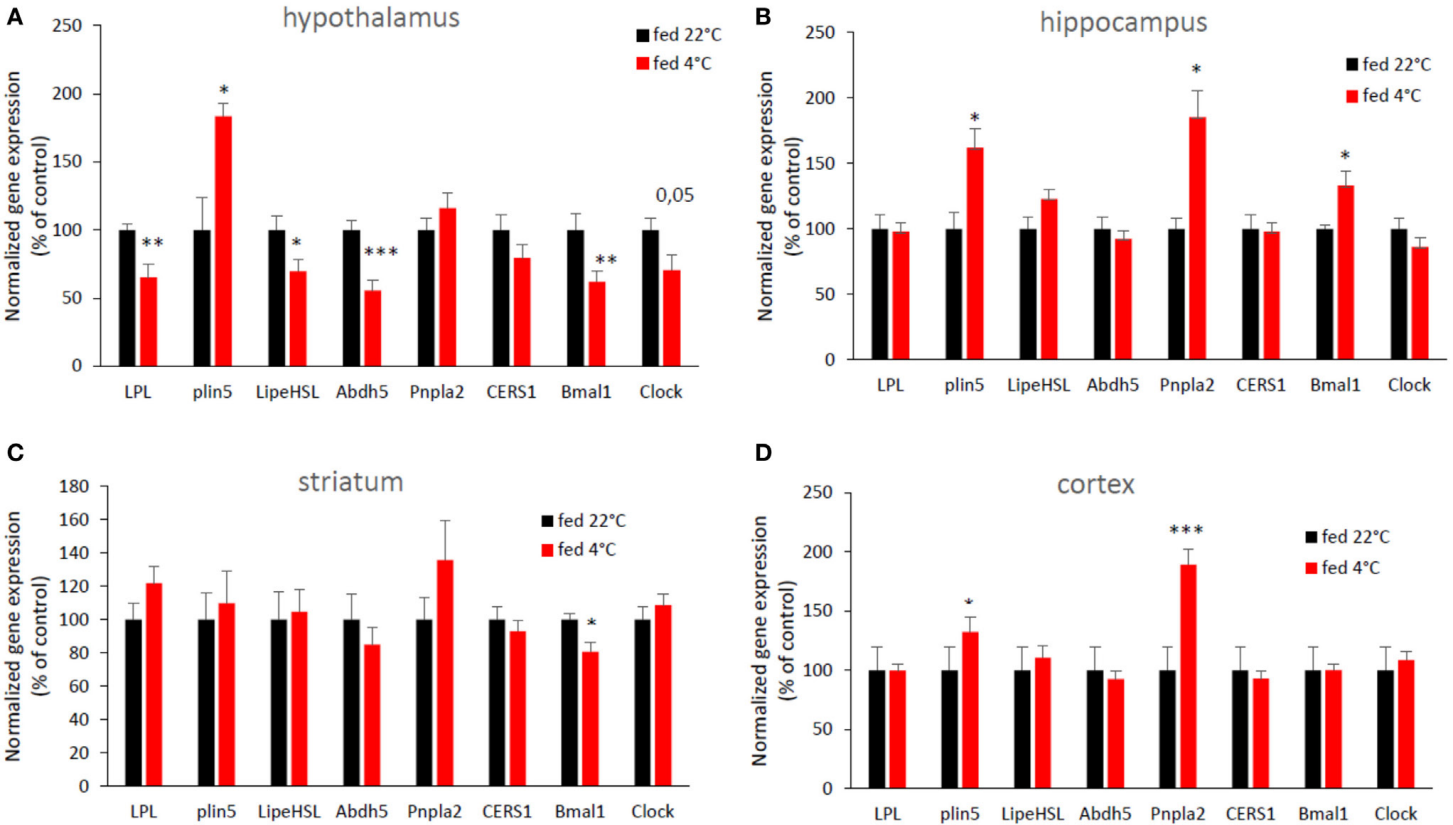
An acute cold exposure modifies the expression of several genes involved in lipid metabolism and circadian clock. mRNA expression in **(A)** hypothalamus, **(B)** hippocampus, **(C)** striatum, and **(D)** cerebral cortex. Data are mean ± SEM and show change of expression rate relative to mRNA for mice at 22°C. *n* = 8–10 for each group. *p < 0.05, **p < 0.01, and ***p < 0.001 vs. “fed 22°C” group.

Cold exposure induced changes in the expression of genes involved in lipid metabolism in several brains areas (Figure 2). LPL mRNA expression was decreased in response to cold exposure in the hypothalamus, as well as hormone sensitive lipase (Lipe HSL) and abhydrolase domain containing 5 (Abhd5) mRNA. Conversely, acute cold exposure significantly increased the expression of hypothalamic perilipin 5 (Plin 5). Abdh5 and Plin5 are cofactors of the Pnpla2 (also known as ATGL) which catalyzes the first step of intracellular TG hydrolysis (16). No change was observed in the expression of other hypothalamic genes involved in lipid metabolism. In the hippocampus and in the cerebral cortex, cold exposure significantly increased the expression of the perilipin 5 gene, as in the hypothalamus, but also of Pnpla2 (ATGL). No change in expression of mRNAs for lipases or their cofactors was seen in the striatum. For clock genes, the expression of hypothalamic Bmal1 and Clock and striatal Bmal1 mRNA was decreased, while hippocampal Bmal1 expression was increased after cold exposure.

### MBH LPL Depletion Dysregulates Body Temperature and Energy Metabolism

**Figure 3:**
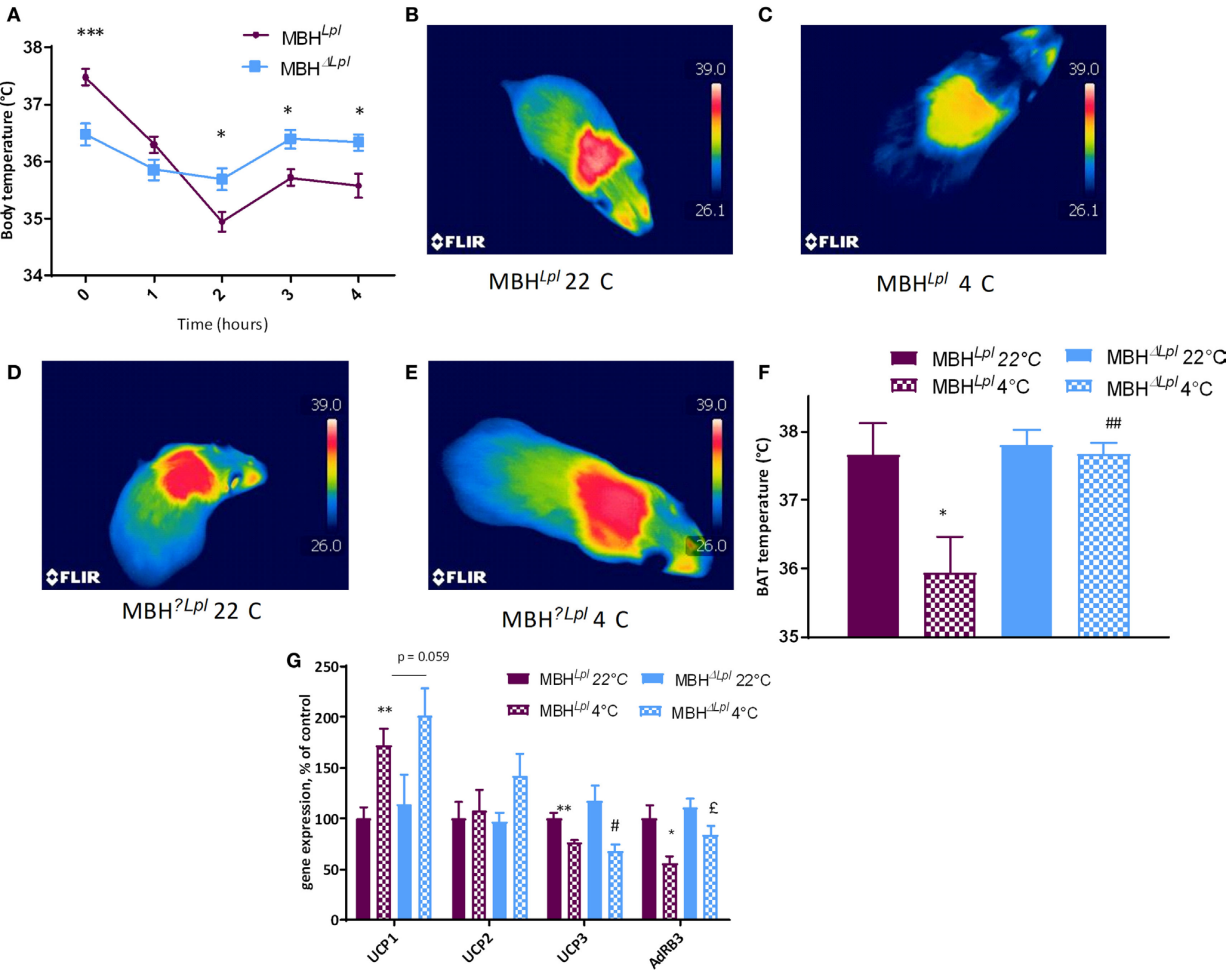
An acute exposure to cold decreases body and skin temperature of MBH*^Lpl^* mice but not of MBHΔ*^Lp^* **(A)**. Thermic images of brown adipose tissue (BAT) temperature of MBH*^Lpl^* at 22°C **(B)** and at 4°C **(C)** shows a decrease during the acute cold exposure whereas BAT temperature of MBHΔ*^Lpl^* at 22°C **(D)**, is maintained at 4°C **(E)**. The value of BAT temperature for MBH*^Lpl^* and MBHΔ^LPL^ mice at 22°C and at 4°C is presented in a global histogram, showing a decrease of BAT temperature at 4°C for MBH*^Lpl^* whereas MBHΔ*^Lpl^* mice present no difference at 4°C **(F)**. Measurement of mRNA expression in BAT **(G)** for uncoupling proteins (UCPs) show no difference between MBH*^Lpl^* and MBHΔ*^Lpl^* at 22 or at 4°C. mRNA expression of AdRβ3 is increased at 4°C in MBHΔ^LPL^ group compared to MBH*^Lpl^*. Data are mean ± SEM (*n* = 6). *p < 0.05, **p < 0.01 vs. MBH*^Lpl^* mice at 22°C, ^£^*p* < 0.05 vs. MBH*^Lpl^* mice at 4°C.

As shown in Table 2 and Figure 3A, MBHΔ*^Lpl^* mice had a significantly lower basal temperature at 22°C when compared with controls mice. However, after 4 h of exposure to 4°C, MBHΔ*^Lpl^* mice maintained their body temperature, whereas body temperature fell by 1.2°C during the first hour of cold exposure in control MBH*^Lpl^* mice and remained lower than that in MBHΔ*^Lpl^* mice for the remainder of the 4 h exposure period. Figure S1 in Supplementary Material shows the temperature time courses for MBH*^Lpl^* and MBHΔ*^Lpl^* mice with their respective controls maintained at 22°C during 4 h. The thermogenic images over intrascapular BAT pads (Figures 3B–E) and their quantification (Figure 3F) appear to correlate with comparable changes in core body temperature measured with the intraperitoneal probes: MBHΔ*^Lpl^* mice maintained their BAT temperature after 4 h at 4°C, whereas BAT temperature significantly dropped in controls. The expression of uncoupling protein 1 (UCP1) mRNA was upregulated by 4 h of cold exposure in both MBH*^Lpl^* and MBHΔ*^Lpl^* mice (Figure 3G), and there was no difference in UCP2 and UCP3 expressions in MBHΔ*^Lpl^* mice compared to controls. In MBH*^Lpl^* mice, expression of β3-adrenoreceptor mRNA was significantly downregulated by the cold exposure, whereas it remained higher in MBHΔ*^Lpl^* mice than their 4°C controls (Figure 3G).

**Table 2:** Comparison of physiological parameters of MBH*^Lpl^* vs. MBHΔ*^Lpl^*.

	MBH*^Lpl^*	MBHΔ*^Lpl^*
Body weight (g)	33.07 ± 0.63	31.45 ± 0.88
Body temperature before cold exposure (°C)	37.02 ± 0.12	36.26 ± 0.10***
Body temperature after cold exposure (°C)	35.58 ± 0.21	36.34 ± 0.15*
Temperature change (°C)	−1.31 ± 0.14	+0.02 ± 0.11***
Weight of BAT at 22°C (g)	0.11 ± 0.026	0.15 ± 0.025
Weight of BAT at 4°C (g)	0.094 ± 0.015	0.16 ± 0.021*

*Data are mean ± SEM, n ≥ 6 for each group*.

*p < 0.05 vs. MBH^Lpl^.

***p < 0.001 vs. MBH^Lpl^.

**Figure 4:**
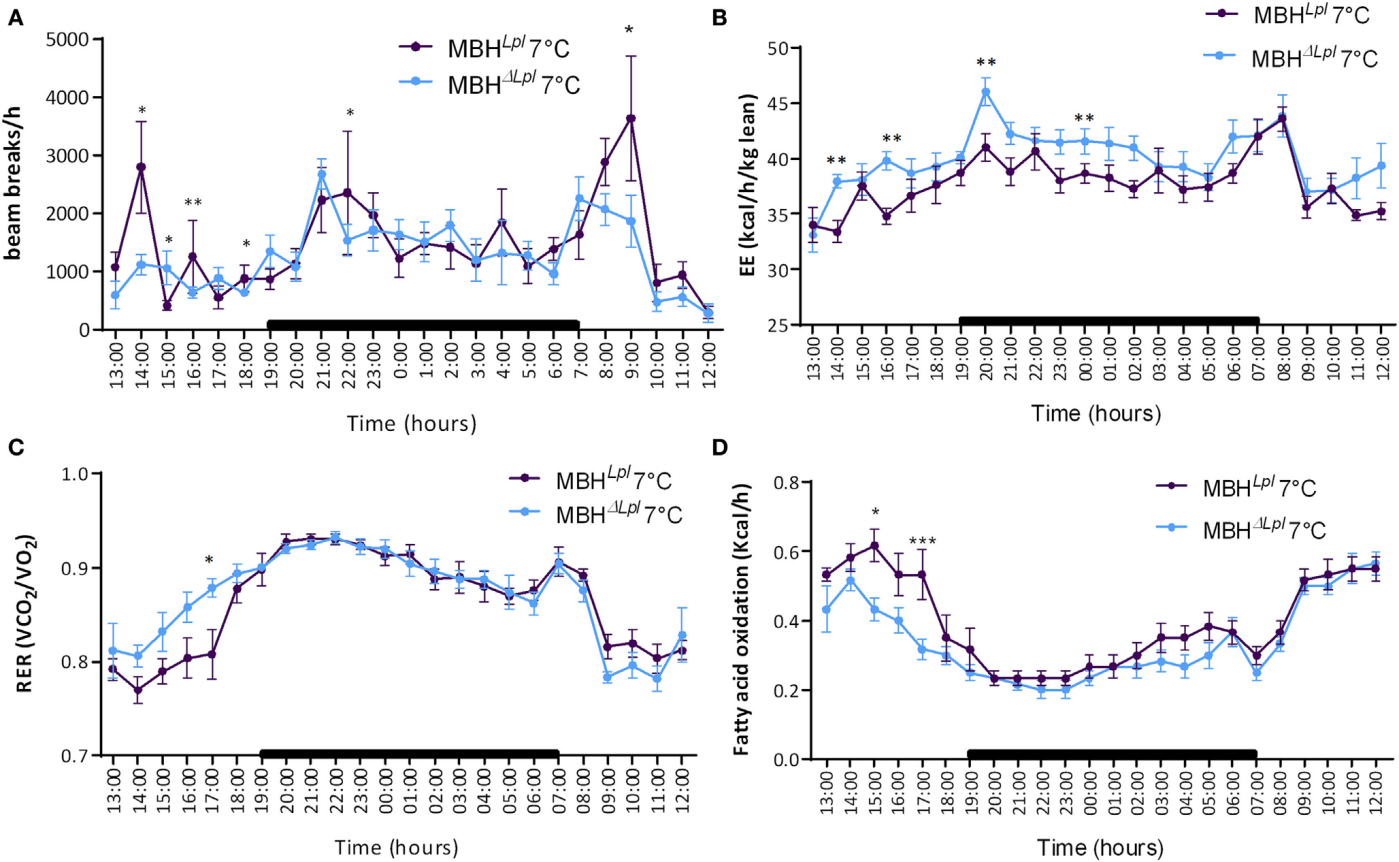
Calorimetry and locomotor activity for MBH*^LPL^* and MBHΔ*^LPL^* mice during 24 h of cold exposure at 7°C. **(A)** Locomotor activity. **(B)** Energy expenditure normalized per lean body mass. **(C)** Respiratory exchange rate (RER, VCO_2_/VO_2_). **(D)** Fatty acids oxidation rate. Data are mean ± SEM (*n* = 5–6). *p < 0.05, **p < 0.01, and ***p < 0.001 vs. MBH*^Lpl^* mice.

During 24 h cold exposure in metabolic cages, food intake was similar in the two groups (Figure S2 in Supplementary Material), and the locomotor activity was decreased during the daylight (cf. Figure 4A) whereas energy expenditure normalized to lean mass was significantly increased in MBHΔ*^Lpl^* mice (Figure 4B). A shown in Figure 4C, these mice presented a trend toward carbohydrate-oriented metabolism before the nighttime, as demonstrated by the increased RER, and consequently a lower rate of fatty acid oxidation (Figure 4D).

### The Expression of “Beige” Genes Is Increased in WAT of MBHΔ^*Lpl*^ Mice

**Figure 5:**
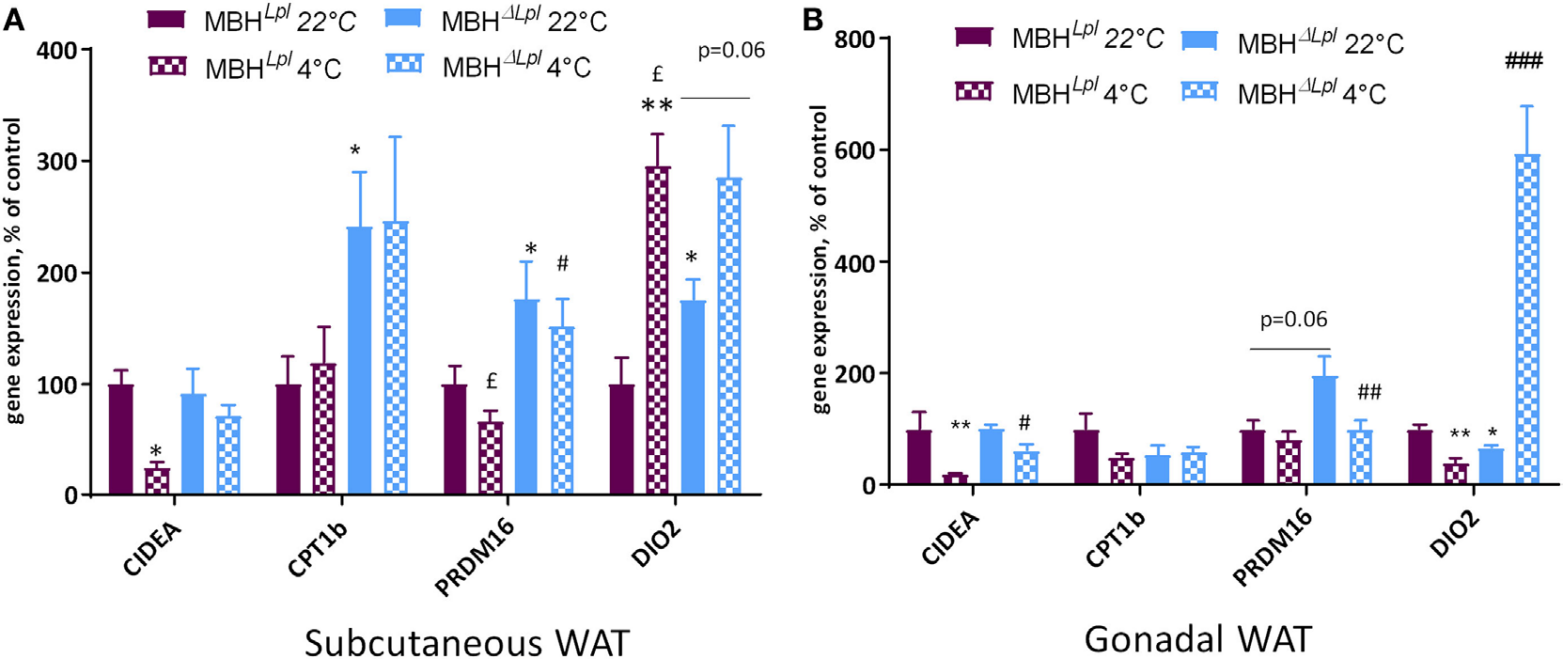
Depletion of lipoprotein lipase in the mediobasal hypothalamus (MBH) increases mRNA expression of genes responsible for “beigeing” in white adipose tissues (WATs). **(A)** mRNA expression in subcutaneous WAT. **(B)** mRNA expression in gonadal WAT. Data are mean ± SEM and show change of expression rate relative to mRNA for mice at 22°C. *n* = 4–6 for each group. *p < 0.05, **p < 0.01 vs. MBH*^Lpl^* mice at 22°C, ^#^*p* < 0.05, ^##^*p* < 0.01, and ^###^*p* < 0.001 vs. MBH*^Lpl^* mice at 4°C, ^£^*p* < 0.05 vs. MBHΔ*^Lpl^* mice at 22°C.

Beige adipocytes share some common characteristics with brown adipocytes and have a thermogenic potential. In the subcutaneous adipose tissue, some genes involved in WAT thermogenic function (“beige” markers; CPT1b and PRDM16) were significantly increased in basal conditions (22°C) and remained higher compared than controls after a cold exposure. Moreover, Dio2 gene expression was higher at 22°C in MBHΔ*^Lpl^* mice (Figure 5A). In gonadal WAT, PRDM6 trended to be more expressed in MBHΔ*^Lpl^* mice compared to controls at 22°C, and DiO2 expression showed a ninefold increase following cold exposure (cf. Figure 5B).

## Discussion

In the present study, we first demonstrated that hypothalamic LPL mRNA expression and activity were regulated as a function of ambient temperatures between 22 and 4^o^C. Indeed, cold exposure induced a selective decrease in both LPL activity and gene expression in hypothalamus with no change in mRNA expression in the hippocampus, striatum, or cerebral cortex when temperature dropped from 22 to 4°C. It has already been reported that the expression of the brain LPL was regulated by the 72 h fasted or fed states (29), but to our knowledge it is the first time that a regulation as a function of the ambient temperature is described. We and others previously reported that brain LPL regulated energy balance. Wang et al. showed that neuronal LPL deficiency promoted obesity and maladaptive responses to environmental challenges (30–32). In addition, we reported that specific deletion of hypothalamic LPL induced body weight gain without hyperphagia, but with increased food efficiency in mice (20). Such increased body weight gain was partly explained by an early decrease in locomotor activity, a trend toward lower energy expenditure at night, and a preferential use of carbohydrate rather than lipid metabolism (20).

In the present study, after having shown that LPL in the hypothalamus was regulated by ambient temperature, it seemed important to investigate whether partial invalidation of LPL in the hypothalamus could therefore influence cold adaptation in mice in order to establish a cause-and-effect relationship between the expression of the enzyme and body temperature. It is well known that the control of BAT thermogenesis is regulated by the CNS (33, 34), and numerous studies have highlighted the role of the hypothalamus and the SNS in such control (4, 27, 35, 36). Besides the preoptic area of the hypothalamus, which is considered as a major coordinator of thermoregulation since it receives inputs from skin and visceral thermoreceptors, other nuclei participate in thermoregulation such as the paraventricular, the ARC and the VMH [review by Labbe et al. (4)]. Although the characterization of the neurons controlling thermogenesis remains unsolved, data pointed toward a role for the melanocortin system in stimulating BAT thermogenesis (3). In the ARC, NPY/AgRP neurons inhibit this system while POMC neurons activate it. In the VMH, steroidogenic factor 1 (SF1) neurons are known to affect BAT thermogenesis (37), by integrating both AMPK and mTOR signaling (27, 38, 39). We showed here that LPL deficiency in the hypothalamus induced a dysregulation of thermogenesis in mice at both normal vivarium temperature of 22 and at 4^o^C. Specifically, MBHΔ*^Lpl^* mice had a significant lower core body temperature compared to controls when the ambient temperature was 22°C. This result seemed to be consistent with our previous observations (i.e., sparing fatty acids and decrease of locomotor activity) (20). As mentioned above, we observed a decrease in both LPL expression and activity in the hypothalamus in normal mice during cold exposure. This result could suggest that the physiological decrease of LPL activity in the hypothalamus, in response to decreased ambient temperature plays a role in adaptive thermogenesis. In accordance with this hypothesis, when the hypothalamic LPL was permanently depleted, the animals presented higher energy expenditure and were able to defend their body temperature more successfully than controls when exposed at 4°C for 4 h. At mild temperature corresponding to their basal housing conditions (22°C), LPL depleted animals displayed a significantly lower body temperature (0.76°C), which could be interpreted as an attempt of saving energy for the long-term. The subsequent question might be to understand how a drop in hypothalamic LPL activity could induce this change in energy flow and control body temperature. It can be hypothesized that a decrease in hypothalamic LPL activity and thus a consequent local decrease in TG hydrolysis and FFA availability could be sensed by specialized neurons (18, 40) as a “thrifty” signal and a need for metabolic adaption. By specifically decreasing LPL in MBH, we therefore mimicked such a “thrifty behavior” that was observed during cold exposure (41). Because MBHΔ*^Lpl^* mice had already displayed some metabolic features of cold exposure, the actual exposure to cold (at least during a short period) did not induce a greater decrease in their body temperature, whereas this occurred in the control mice (decreased body temperature from 37 to 35°C). This adaption could be explained by a modulation of the circadian clock, as it has been shown that transcription of genes involved in the metabolism can be regulated by the circadian clock. For example, Clock and Bmal1 genes favor transcription of Lpl (42). Interestingly, we showed that exposure to cold decreased the expression of Bmal1 and Clock. The downregulation of Bmal1 and Clock mRNA could partly explain the decrease observed for LPL mRNA. Furthermore, a recent study suggested the existence of an independent circadian clock in the VMH (43). Exposure to cold could have an impact on hypothalamic circadian clock and thus on LPL expression. Finally, this study by Orozco-Solis et al. showed that a mouse model KO for Bmal1 in Sf1 neurons of the VMH was associated with increased BAT temperature throughout the diurnal cycle. Therefore, a decrease of Bmal1 expression during cold exposure in MBHΔ*^Lpl^* compared to MBH*^Lpl^* could contribute to explain the preservation of BAT temperature.

Partial deletion of hypothalamic LPL also resulted in changes in mRNA expression of some marker genes of adipocyte function as well as thermogenesis-related genes. We measured some of these genes of interest in subcutaneous and gonadal adipose tissues. Interestingly, MBHΔ*^Lp^*^l^ mice that were exposed to mild temperature showed a significant increase in PRDM16 expression in WATsc and a tendency in WATgon when compared with controls. PRDM16 is a critical transcriptional regulator of thermogenesis and the increase of PRDM16 in WATsc of MBHΔ*^Lpl^* mice could be interpreted as an attempt to adapt to their decreased body temperature at an ambient temperature of 22°C. It has been reported that adipocyte-specific deletion of PRDM16 markedly inhibited beige adipocyte function in subcutaneous fat following cold exposure or β3-agonist treatment (44). Thus, browning may occur in WATsc of MBHΔ*^Lpl^* mice as an adaptation to chronically lowered body temperature. However, it must be pointed out that such adaptation was not sufficient to bring the body temperature back to that observed in the control mice. This can be explained by the fact that other parameters were also affected by the hypothalamic deletion of LPL (20).

Interestingly, mRNA expression of Dio2, the enzyme that activates T4 to 3,3′,5-triiodothyronine (T3) was also significantly elevated in WATsc of MBHΔ*^Lpl^* mice compared to controls exposed to 22°C. Dio2 is also known as being a thermogenesis-related gene (9). As for PRDM16, the local increase in Dio2 mRNA could be interpreted as an adaptation to the decrease in body temperature through increased local production of T3. During cold exposure, mRNA expression of Dio2 in WATsc was significantly increased in controls to reach a value similar to MBHΔ*^Lp^* mice. As mentioned, exposure to cold induced a decrease in the body temperature of control mice (from 37 to 35°C) while that of MBHΔ*^LPL^* mice remained constant compared to exposure to mild. During cold exposure, PRDM16 expression remained significantly higher in WATsc of MBHΔ*^LPL^* mice compared to controls. In WATgon, Dio2 was significantly upregulated in MBHΔ*^Lp^* mice exposed to 4°C as compared with their controls. This could be related to the fact that they managed to maintain their body temperature: the upregulation of browning markers, already present at 22°C, may in part explain the absence of significant fluctuation in body temperature during exposure to cold. In particular, de Jesus et al. showed that increased Dio2 expression in BAT played a key role for local production of T3, even in a situation where serum concentrations of both T3 and T4 remained constant during cold stress (24 h at 4°C) (7). Such a result highlighted a key role for local production of T3 through activation of Dio2.

Finally, skin temperature surrounding BAT was recorded as an indirect index of thermogenesis and BAT function. In control mice, skin temperature decreased when mice were exposed to cold whereas it remained unchanged compared to mild temperature exposure for MBHΔ*^Lpl^*. This could be related to the decrease in body temperature that we also observed. However, in both groups UCP1 mRNA expression was increased during cold temperature when compared to mild exposure. Again, this difference in response between the two groups in the face of cold exposure suggests that the deficiency of LPL in the hypothalamus regulated energy homeostasis and especially thermogenesis.

In conclusion, we demonstrate here for the first time, that LPL in hypothalamus is a negative regulator of thermogenesis. Normal adaptation to a drop-in temperature involves a decrease in hypothalamic LPL activity that promotes cold-induced thermogenesis. A chronic decreased in LPL activity in hypothalamus could impair the regulation of body temperature in response to changes in ambient temperature. More generally, a defect in the central sensing of lipids could compromise the survival of individuals during winter and more particularly when a lack of food also occurred.

## Ethics Statement

The experimental protocol was approved by the institutional animal care and use committee of the Paris Diderot University (CEEA40), and the agreement # 03752.02 was given to the project.

## Author Contributions

ML, SL, CC-G, and CM designed experiments. EL, RD, NK, CC, and CC-G performed experiments. EL, CC-G, and CM prepared the manuscript. RD, CC, and ML edited the manuscript. The entire study was supervised by CC-G and CM.

## Conflict of Interest Statement

The authors declare that the research was conducted in the absence of any commercial or financial relationships that could be construed as a potential conflict of interest.
