# Supplementary material for: Lipoprotein Lipase Expression in Hypothalamus Is Involved in the Central Regulation of Thermogenesis and the Response to Cold Exposure
Source: Front Endocrinol (Lausanne). 2018 Mar 14;9:103. doi: 10.3389/fendo.2018.00103 (PMC5861133; doi:10.3389/fendo.2018.00103)
Supplement: Supplementary file 1 [file Image_1.PDF]

A

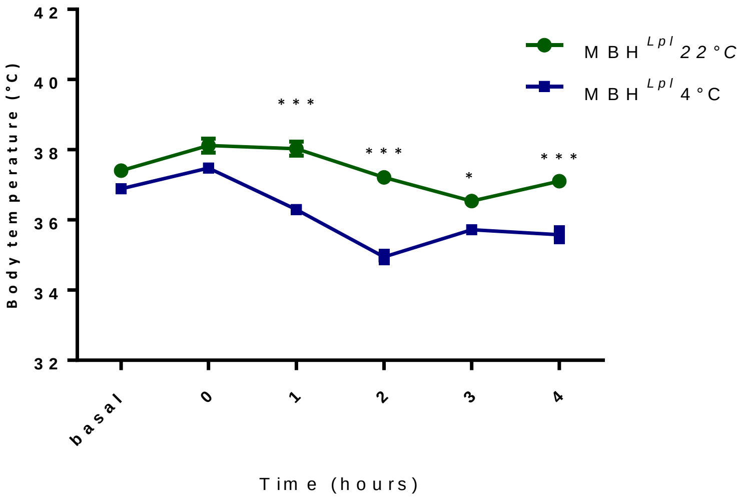

B

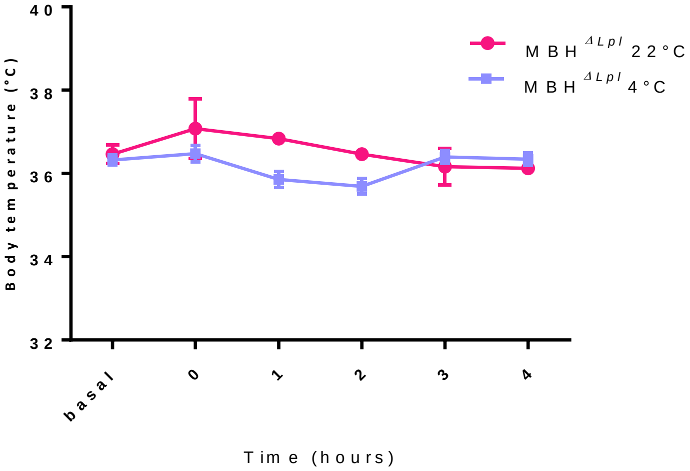

Supplemental Figure 1. Temperature time courses for mice during exposure to cold (4hours at 4°C, starting at Time 0) compared with their respective controls maintained to 22°C . (A) Body temperature of MBH^{Lpl} at 22°C vs. MBH^{Lpl} at 4°C. (B) Body temperature of MBH$^{\Delta Lpl}$ at 22°C vs. MBH^{Lpl} at 4°C. n=6 for each group. ***$p < 0.001$ vs MBH^{Lpl} mice at 22°C.

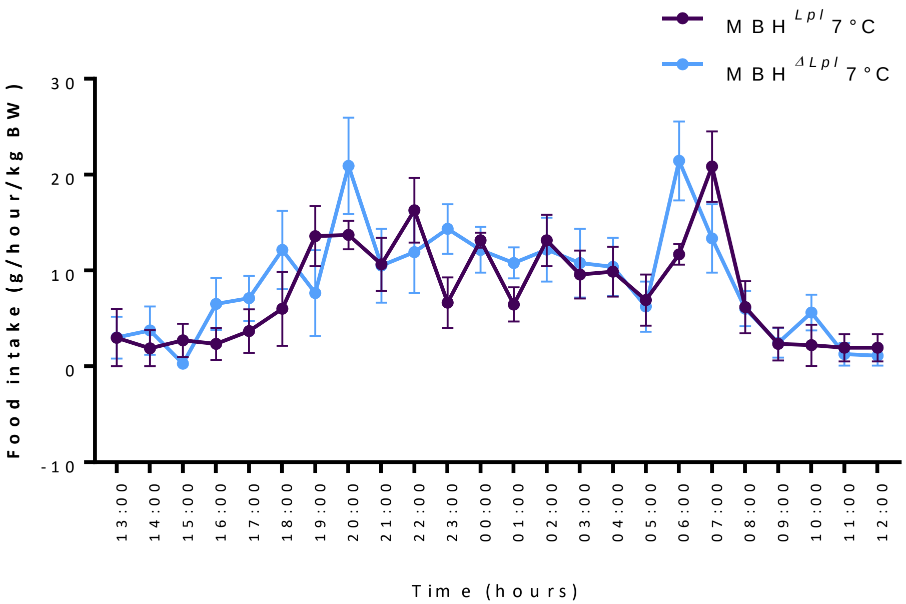

Supplemental Figure 2. Measurement of food intake of MBH^{Lpl} vs. MBH$^{\Delta Lpl}$ during exposure to 7°C for 24 hours. There is no difference between the two groups. n=6 for each group.
